# Case report: Identification of facioscapulohumeral muscular dystrophy 1 in two siblings with normal phenotypic parents using optical genome mapping

**DOI:** 10.3389/fneur.2024.1258831

**Published:** 2024-02-01

**Authors:** Jieni Jiang, Xiaotang Cai, Haibo Qu, Qiang Yao, Tiantian He, Mei Yang, Hui Zhou, Xuemei Zhang

**Affiliations:** ^1^Department of Medical Genetics and Prenatal Diagnosis Center, West China Second University Hospital, Sichuan University, Chengdu, China; ^2^Department of Obstetrics and Gynecology, West China Second University Hospital, Sichuan University, Chengdu, China; ^3^Key Laboratory of Birth Defects and Related Diseases of Women and Children (Sichuan University), Ministry of Education, Chengdu, China; ^4^Department of Rehabilitation, West China Second University Hospital, Sichuan University, Chengdu, China; ^5^Department of Radiology, West China Second University Hospital, Sichuan University, Chengdu, China

**Keywords:** facioscapulohumeral muscular dystrophy 1, optical genome mapping, sibling patient, low-level mosaicism, germline mosaicism, disease penetrance, molecular diagnosis, methylation analysis

## Abstract

**Objective:**

Facioscapulohumeral muscular dystrophy type 1 (FSHD1) is one of the most common forms of autosomal-dominant muscular dystrophies characterized by variable disease penetrance due to shortened D4Z4 repeat units on 4q35. The molecular diagnosis of FSHD1 is usually made by Southern blotting, which is complex, time-consuming, and lacks clinical practicality. Therefore, in this study, optical genome mapping (OGM) is employed for the genetic diagnosis of FSHD1. Furthermore, epigenetic heterogeneity is determined from methylation analysis.

**Methods:**

Genomic DNA samples from four members of the same family were subjected to whole-exome sequencing. OGM was used to identify structural variations in D4Z4, while sodium bisulfite sequencing helped identify the methylation levels of CpG sites in a region located distally to the D4Z4 array. A multidisciplinary team collected the clinical data, and comprehensive family analyses aided in the assessment of phenotypes and genotypes.

**Results:**

Whole-exome sequencing did not reveal variants related to clinical phenotypes in the patients. OGM showed that the proband was a compound heterozygote for the 4qA allele with four and eight D4Z4 repeat units, whereas the affected younger brother had only one 4qA allele with four D4Z4 repeat units. Both the proband and her younger brother were found to display asymmetric weakness predominantly involving the facial, shoulder girdle, and upper arm muscles, whereas the younger brother had more severe clinical symptoms. The proband's father, who was found to be normal after a neurological examination, also carried the 4qA allele with eight D4Z4 repeat units. The unaffected mother exhibited 49 D4Z4 repeat units of the 4qA allele and a minor mosaic pattern with four D4Z4 repeat units of the 4qA allele. Consequently, the presence of the 4qA allele in the four D4Z4 repeat units strongly pointed to the occurrence of maternal germline mosaicism. The CpG6 methylation levels were lower in symptomatic patients compared to those in the asymptomatic parents. The older sister had lower clinical scores and ACSS and higher CpG6 methylation levels than that of her younger brother.

**Conclusions:**

In this study, two siblings with FSHD1 with phenotypically normal parents were identified by OGM. Our findings suggest that the 4qA allele of four D4Z4 repeats was inherited through maternal germline mosaicism. The clinical phenotype heterogeneity is influenced by the CpG6 methylation levels. The results of this study greatly aid in the molecular diagnosis of FSHD1 and in also understanding the clinical phenotypic variability underlying the disease.

## Introduction

Facioscapulohumeral muscular dystrophy (FSHD; OMIM 158900), which is one of the most common hereditary myopathies, is predominantly characterized by progressive asymmetrical weakness of the facial and shoulder girdle muscles. The severity of the disease varies with age and sex. Thus, the degree of muscle damage shows a vast difference at the clinical level, ranging from almost asymptomatic weakness related to eye closure to significant disability with weakness of the shoulder and pelvic girdle and bilateral leg drop (1, 2). These clinical manifestations are characteristic of the highly heterogeneous nature of the FSHD phenotype.

The genetic changes associated with FSHD are highly complicated, and the disease's origin is considered to be commonly linked to the disruption of epigenetic silencing mechanisms, which leads to the abnormal expression of the distal double homeobox protein 4 (*DUX4*) gene. Type I FSHD is characterized by a macrosatellite array of tandem D4Z4 repeat units at the distal end of chromosomal region 4q35 (3), as well as a permissive type-A haplotype after the distal repeat that maintains stable *DUX4* transcription (4). FSHD1, the most common form of FSHD, is the most frequent finding as it is diagnosed in 95% of all clinical cases presented and is found to be caused by an autosomal-dominant route. The 1–10 D4Z4 units are repeated in individuals with a pathogenic form of FSHD1, whereas 11–150 repeat units are present in unaffected individuals (5). Another pathogenic form, FSHD2, is caused by mutated epigenetic modifiers, such as *SMCHD1* or *DNMT3B* (6, 7), as a result of digenic inheritance.

The molecular diagnosis of FSHD1 is made based on the number of D4Z4 repeat units at the chromosome 4q35 locus in the presence of a permissive 4q35A haplotype. But this diagnosis is complicated by the size of the repeat units, which have an approximate length of 3.3 kb, and their variable number. In addition, homologous polymorphic repeat arrays are present on chromosomes 4 and 10, and there is a possible exchange between these chromosomal regions. These factors enable the molecular diagnosis of FSHD. The traditional genetic methods employed for diagnosing FSHD are pulsed-field gel electrophoresis and Southern blotting (8). However, both these techniques are complex and time-consuming, require a large amount of high-quality DNA, and they cannot be used to accurately determine critical lengths (9, 10). A simple and uniform method of diagnosis that enables easy interpretation replication by laboratories worldwide would be beneficial. Optical genome mapping (OGM) offers an accurate and highly reproducible method for identifying FSHD-associated chromosomal abnormalities (11). This method overcomes some of the important problems encountered in conventional analytical techniques, such as distinguishing 4q35-D4Z4 repeats from the highly homologous 10q26 array, measuring the number of repeats at 4q35, and differentiating between the 4qA and 4qB alleles (12).

Previous studies have suggested that reduced DNA methylation levels in potentially pathogenic alleles could be used as a reliable marker for diagnosing FSHD (13). Furthermore, various clinical features observed in FSHD, such as penetrance variability, gender bias in severity, and asymmetric muscle wasting, can be explained by reduced methylation.

In this study, we employed OGM for the diagnosis of FSHD by identifying D4Z4 repeat units within permissive 4qA haplotypes. We complemented this diagnosis by examining the CpG methylation levels within the furthest D4Z4 arrays, which is used as a particularly critical approach for diagnosing, predicting, and providing genetic counseling to individuals carrying D4Z4 alleles of borderline size and their relatives with the same reduced D4Z4 allele.

## Patients and methods

### Subjects

Two siblings (the proband and her younger brother) and their unaffected parents were recruited from the West China Second University Hospital (Sichuan University). Their clinical data was recorded by a multidisciplinary team. This study was approved by the Medical Ethics Committee of West China Second University Hospital (Sichuan University). Informed consent was obtained from all study participants.

### Clinical assessments

Clinical data, such as patient history, systemic features, and physical and auxiliary examinations, were collected prospectively. FSHD clinical score (CS) was employed to assess muscle strength. The clinical severity score (CSS) was used to determine the disease severity. The CSSs ranged from 0 to 5, with 10 levels. A score of 0 represented no symptoms of muscle weakness and a score of 5 represented wheelchair-dependent patients with a severe disease. The score increased in increments of 0.5 with a concomitant progress in disease severity. The CSS was adjusted according to patient age at examination to derive the age-corrected clinical severity score (ACSS), which was used to determine disease severity and was calculated as described previously (14, 15):


$$\begin{array}{l} ACSS=\left(\left[CSS\times 2\right]/age\;at\;examination\right)\times \;1,000 \end{array}$$

The clinical features and assessments of the siblings are presented in Table 1.

**Table 1:** Clinical features and assessments of the siblings.

**Subjects**	**III-9**	**III-10**	**II-7**	**II-8**
Sex	F	M	M	F
Relationship	Daughter (proband)	Son	Father	Mother
Age at onset (years)	17	13	/	/
Age at examination(years)	17	15	/	/
Age at diagnosis(years)	27	25	50	47
Age at reported(years)	28	26	51	48
**Genetic/epigenetic**				
4q haplotype and D4Z4 repeats units	4qA with 4 D4Z4 repeats, 4qA with 8 D4Z4 repeats	4qA with 4 D4Z4 repeats, 4qB with 28 D4Z4 repeats	4qA with 8 D4Z4 repeats, 4qB with 28 D4Z4 repeats	4qA with 49 D4Z4 repeats and low-level mosaicism of 4 D4Z4 repeats
10q haplotype and D4Z4 repeats units	10qA with 17 D4Z4 repeats/ 10qB with 9 D4Z4 repeats	10qA with 17 D4Z4 repeats	10qA with 17 D4Z4 repeats/ 10qB with 9 D4Z4 repeats	10qA with 7 and 17 D4Z4 repeats
CpG6 methylation values, %	60	20	90	90
**Clinical phenotype**				
Hearing loss	No	No	No	No
Vision loss	No	No	No	No
Dysphagia	No	No	No	No
Impaired intellectual development	No	No	No	No
Facial muscle weakness and atrophy	Severe	Severe	No	No
Shoulder girdle muscle weakness and atrophy	Moderate	Severe	No	No
Upper arm muscle weakness and atrophy	Moderate	Severe	No	No
Abdominal wall muscle weakness and atrophy	No	Severe	No	No
Lower limb and pelvic muscle weakness and atrophy	Mild	Severe	No	No
Foot extensor muscle weakness	No	Severe	No	No
Scapular winging	Yes	Yes	No	No
Spine deformities	Mild	Severe	No	No
Stand up from a chair	Yes, without support	Yes, with support	Yes, without support	Yes, without support
Wheelchair dependency	No	No	No	No
**Assessments, range**				
FSHD clinical score(0-15)	8	9	0	0
Clinical severity score (0–5)	3	4	0	0
Age-corrected clinical severity score (0–10000)	353	533	0	0
**Auxiliary examination**				
Serum creatine kinase(normal range:24-194IU/L)	840 IU/L (in 2012 )	1153 IU/L (in 2012 )	ND	ND
Electromyography	Myogenic changes (in 2012 )	Myogenic changes (in 2012 )	ND	ND
Biopsy of skeletal muscle	ND	Myogenic changes (in 2012)	ND	ND
Echocardiography	No abnormality (in 2022)	No abnormality (in 2022)	ND	ND
X-ray	Slightly scoliotic spine and a straightened cervical spine (in 2022)	Increased physiological curvature of the lumbar spine, and an upturned sacrum. Slight curvature of the spine, a straight cervical spine (in 2022)	ND	ND
MRI	Myogenic changes in the bilateral buttocks and thighs, as well as the right upper arm and paravertebral muscles (in 2022)	ND	ND	ND
Pulmonary function test	Slight decrease in pulmonary ventilation reserve function and mild restrictive ventilatory dysfunction (pulmonary ventilation reserve was 88.1%) (in 2023)	ND	ND	ND
DMD test	Negative (in 2015)	Negative (in 2015)	ND	ND
WES	Negative (in 2021 and 2022)	Negative (in 2018 and 2022)	Negative	Negative

ND, not done.

### Genetic analysis

#### Whole-exome sequencing analysis

Blood samples (2–3 mL) were collected in ethylenediaminetetraacetic acid (EDTA) tubes. Genomic DNA was extracted from all samples using a QIAamp DNA Blood Mini Kit (Qiagen, Valencia, CA, USA), following the manufacturer's guidelines. A whole-exome sequencing (WES) library was done using the NanoWES Human Exome V1Kit (Berry Genomics, Beijing). Sequencing was performed using the NovaSeq6000 platform with 150-base pair (bp) paired-end reads. The mean depth of coverage of the sequenced sample was 100 × . The Burrows–Wheeler aligner software tool was used for aligning the sequencing reads with hg38/GRCh38, and local alignment and recalibration of the base quality of the aligned reads was performed using the GATK Indel Realigner and the GATK Base Recalibrator, respectively (broadinstitute.org/). Single-nucleotide variants (SNVs) and small insertions or deletions were identified using the GATK Unified Genotyper (broadinstitute.org/), and functional annotation was performed using ANNOVAR and the Enliven Variants Annotation Interpretation System (Berry genomics, Beijing). Several public databases were accessed for genome filtering, including gnomAD (http://gnomad.broadinstitute.org/) and the 1000 Genomes Project (http://browser.1000genomes.org). The pathogenicity of the detected SNVs was evaluated based on scientific and medical literature and disease databases, including OMIM (http://www.omim.org), PubMed (https://www.ncbi.nlm.nih.gov/pubmed/), ClinVar (http://www.ncbi.nlm.nih.gov/clinvar), and the Human Gene Mutation Database (http://www.hgmd.org). Variants were classified according to the American College of Medical Genetics and Genomics guidelines. The potential pathogenic variants were validated using Sanger sequencing on an ABI 3500 Genetic Analyzer (Applied Biosystems, Waltham, MA, USA), and the data were evaluated using Chromas software (2.6.5).

#### OGM analysis

Blood samples (2–3 mL) were collected in EDTA tubes, and ultra-high molecular weight (UHMW) DNA was extracted, labeled, and processed for use on the Bionano Genomics Saphyr Platform (Bionano Genomics; San Diego, CA), according to the manufacturer's protocol. UHMW DNA was extracted using the Bionano Prep SP Blood and Cell DNA Isolation Kit (Bionano Genomics). Briefly, cells were treated with a lysis-and-binding buffer to extract gDNA, which was subsequently bound to a nanobind disk. The disk was then washed and eluted in the provided elution buffer. The integrity and size of the isolated DNA were validated using pulsed-field gel electrophoresis. HMW DNA quantification was performed with Qubit dsDNA assay BR kits using a Qubit 2.0 Fluorometer (Thermo Fisher Scientific); the final UHMW DNA concentration was 36–150 ng/μL. To generate the Saphyr data, 750 ng of UHMW DNA was labeled with DLE-green fluorophores at a specific six-base sequence (CTTAAG motif) using the Bionano Prep DLS (Direct Label and Stain) Kit (Bionano) following the manufacturer's protocol. Subsequently, the direct labeling enzyme was digested with Proteinase K (Qiagen), and unbound DL-Green fluorophores were washed using the membrane-adsorption procedure. Then, the UHMW DNA samples were stained blue using DNA stain and quantified using the Qubit^®^ HS (High Sensitivity) dsDNA Assay Kit (Thermo Fisher). The samples were placed in the Saphyr system to capture the images of the labeled DNA molecules. Following the conversion of the images into digital representations of the molecules accompanied by labels, the resulting digital data was transferred to the Bionano Access software. This software functioned as a central data hub, enabling data visualization and facilitating the initiation of secondary analysis. Data generated by Saphyr is automatically assembled in the software Bionano Solve 3.7 and visual analysis is performed using Bionano Access 1.7.

The Bionano EnFocus^TM^ FSHD Analysis pipeline was used to identify the FSHD haplotype and the number of D4Z4 repeat units in patients suspected of FSHD. The pipeline first distinguished the D4Z4 region of chromosome 4 from that of chromosome 10 based on the fluorescent pattern of markers proximal to the D4Z4 repeat region. Then the selected molecules that aligned with these regions were identified so as to create a local assembly of the D4Z4 regions of chromosomes 4 and 10. Subsequently, the resulting genome maps were analyzed to determine the size of the repeat units and assign haplotypes to the alleles. While the DLE-1 enzyme did not directly mark individual D4Z4 units, the FSHD pipeline could estimate the lengths of repeat arrays by analyzing the intervals between labels that bordered the D4Z4 arrays. The pipeline also identified other structural variants and copy-number gains or losses near the D4Z4 repeat unit on chromosome 4 and the SMCHD1 gene on chromosome 18. Additionally, the OGM *de novo* pipeline was employed to assemble comprehensive maps to manually determine the number of large repeat units and haplotypes. To evaluate the quality of the assembled map, the pipeline was used to examine genomic regions that were considered stable according to the hg38 reference genome. To ensure that the molecular quality was good enough for downstream analyses, the quality control criteria were set to an N50 value of ≥200 kbp, a mapping rate of ≥70%, an average label density of 14–17, an effective coverage of ≥75 ×, a positive label variance of 3–10%, and a negative label variance of 6–15% (16).

#### Sodium bisulfite sequencing and CpG methylation analysis

To analyze the methylation levels in CpGs in a region distal to the D4Z4 array in this family, sodium bisulfite sequencing (conducted by Tsingke Biotechnologies, China) was performed with a 4qA allele-specific FasPAS primer, and 10 CpGs were analyzed according to the previously described protocols (17).

## Results

### Clinical presentation

#### Patient 1

**Figure 1:**
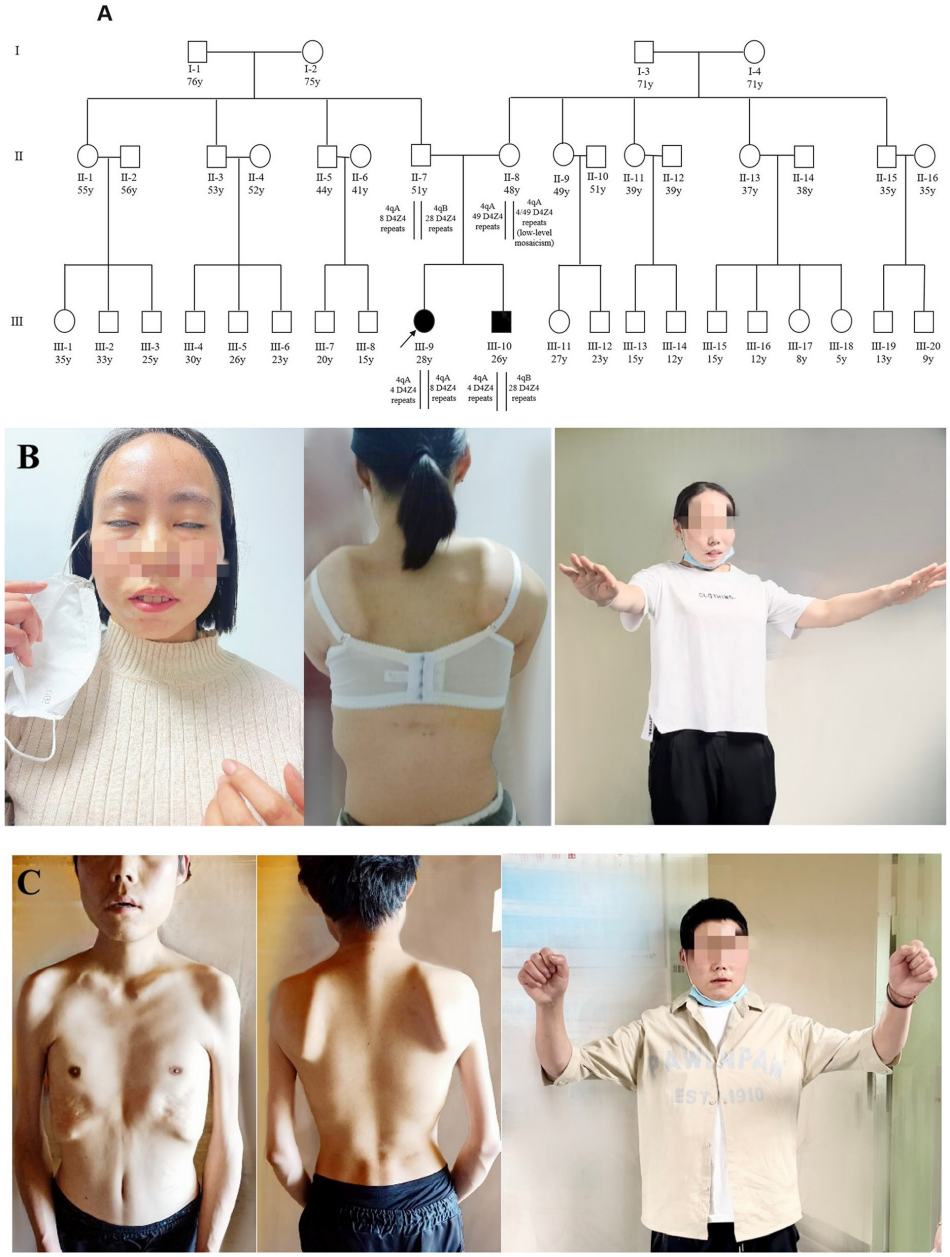
Pedigree and clinical features of two patients. **(A)** Pedigree of the family with FSHD1. The age in years, number of D4Z4 repeat units, and haplotype are shown for each subject. Subject III-9 was identified as a compound heterozygote because of a shortened FSHD1 alleles, whereas her affected brother (III-10) carried just one allele. The parents (II-7 and II-8) were asymptomatic; the father was a heterozygote for eight units of D4Z4 with 4qA, while the mother was a low-level mosaic for four units of D4Z4 with 4qA. All other members of this family were asymptomatic. **(B)** The proband (III-9) showed decreased facial expression and an inability to close her eyes completely (left). She showed false hypertrophy of the orbicularis oris, her lips were thickened and slightly distorted (left), and she presented with a winged scapula (middle) and limitations in lifting activities in the bilateral upper limbs (right). **(C)** Patient 2 (III-10) showed atrophy of the facial muscles (middle), shoulder, upper arm, and chest muscles (right). He presented with a winged scapula (middle) and limitations in lifting activities in the bilateral upper limbs (right). The photographs of the proband (III-9) and the proband's younger brother (III-10) were taken at ages 27 and 25, respectively.

The proband (patient III-9, Figure 1A), a 27-year-old married woman with undiagnosed myopathy, was referred to our hospital for the confirmation of facioscapulohumeral muscular dystrophy type 1 and an assessment of pregnancy risk. She was the first child born to non-consanguineous parents. Neither of the parents presented with any relevant medical history or neuromuscular symptoms (Supplementary Figure 1). At the age of 17, when her younger brother (aged 15) visited a hospital for a possible diagnosis of muscular atrophy after experiencing muscle weakness in his bilateral upper limbs for 2 years, she realized that she also had symptoms similar to those of her younger brother, which presented in her right upper arm, with slightly limited lifting activities. Her creatine kinase level was 840 IU/L (normal range: 24–194 IU/L). Electromyographic analysis revealed myogenic changes in both the lower limbs and a physician at a local hospital suspected the condition to be muscular dystrophy. The symptoms did not improve significantly after the rehabilitation treatment. Three years later (at the age of 20), the symptoms of muscle atrophy in her upper arms were significantly aggravated, and she had difficulty in climbling stairs, running, and jumping. By the time she was 27, she obviously had limited lifting ability in her upper arms. Though she could take care of herself, she had difficulty in combing her hair. She was still able to live independently but could not perform activities that demanded heavy physical work. Her FSHD CS (8) and ACSS (353) are shown in Table 1.

Upon physical examination (in 2022), the patient displayed limited facial expression and could not close her eyes at all (Figure 1B). Her lips were thickened and slightly distorted with false hypertrophy of the orbicularis oris, which made it difficult for her to purse her lips, and she could not puff her cheeks or whistle. She had asymmetric muscle weakness in the upper arm, a clear “winged scapula” presentation (Figure 1B) when her arms were extended forward, and demonstrated a “duck stance” when running. She exhibited a limited range of motion when extending both arms to the sides or front but had no involuntary movements, and her deep tendon reflexes were normal. Her sensation, coordination, and cognitive functions were normal, and she had no hearing loss or significant visual impairment. No scoliosis or apparent abnormalities in squatting or gait were observed.

**Figure 2:**
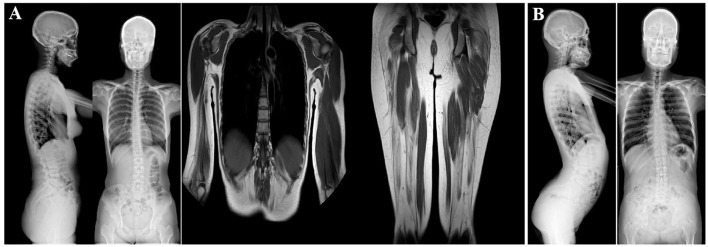
Imagological examination of two patients. **(A)** X-ray examination of patient 1 (III-9). She had a slightly scoliotic spine and a straightened cervical spine **(left)**. MRI analysis of the limb muscles revealed changes in the bilateral buttocks and thighs and the right upper arm and paravertebral muscles (middle and right). These changes were consistent with the presence of muscular dystrophy. **(B)** X-ray examination of the spine of patient 2 (III-10). The left image shows increased physiological curvature of the lumbar spine and an upturned sacrum. The right image shows a slight curvature of the spine, a straight cervical spine.

Radiographic examination (in 2022) of the spine revealed a slight curvature, and the cervical spine was found to be straightened. Limb-muscle magnetic-resonance imaging (MRI) analysis (in 2022) showed changes in the bilateral buttocks and thighs, as well as in the right upper arm and paravertebral muscles (Figure 2A). These changes were consistent with muscular dystrophy. Multiplex ligation-dependent probe amplification (MLPA) did not highlight deletions and duplications in the *DMD* gene (in 2015). WES was performed at another hospital, and the results were also negative (in 2021).

Additional tests were performed to assess risks associated with pregnancy (in 2022). Pulmonary function tests showed a slight decrease in pulmonary ventilation reserve function and mild restrictive ventilatory dysfunction (pulmonary ventilation reserve, 88.1%). A 24-h Holter electrocardiogram revealed sinus arrhythmia. Color Doppler echocardiography showed no abnormalities, whereas cardiac MRI revealed slightly dense muscle trabeculae in the left ventricle. However, the left ventricular systolic function was normal.

#### Patient 2

The proband's younger brother (patient III-10, Figure 1A), was 25 years old when he began participating in this case study. He first noticed symptoms of muscular atrophy of the bilateral upper arms when he was 13 years old without any apparent cause but did not pay attention to it. At the age of 15 years, the symptoms gradually worsened, with mild limitations in lifting activities, and his creatine kinase level was 1,153 IU/L (normal value: 24–194 IU/L). Electromyographic analysis showed myogenic changes in the bilateral upper and right lower limb muscles. A muscle biopsy in the right upper extremity revealed primary muscular dystrophy. He was suspected of having myotonic dystrophy, but his symptoms did not improve significantly after a rehabilitation treatment. His symptoms worsened with each passing year, and the muscles of both shoulders, upper arms, and chest began to atrophy. At the age of 25 years, he and his older sister visited our hospital for confirmation of the disease. Currently, he has weakness in the muscles of the upper limbs, facial weakness, an abnormal gait when walking, difficulty standing after squatting, and more severe clinical symptoms than those of his sister. His FSHD CS (9) and ACSS (533) were higher than those of his older sister (8 and 353, respectively), as shown in Table 1.

Upon physical examination (in 2022), he displayed no movements in his facial expression and could not close his eyes completely; his lips were thickened and slightly distorted with false hypertrophy of the orbicularis oris, and he could not puff his cheeks or whistle. The patient showed substantially winged scapula and limited shoulder girdle mobility. He was unable to raise his arms above his shoulders (Figure 1C). He struggled to rise from a squatting to a standing position and walked with a waddling gait. He also had significant bilaterally winged scapulae, which were more severe than those of the proband. He exhibited limited extension of both upper limbs to the sides and front with no signs of involuntary movement. His sensation, coordination, and cognitive functions were normal. No hearing loss or visual impairments were observed.

Radiographic analysis (in 2022) revealed a slight curvature of the spine, a straight cervical spine, increased physiological curvature of the lumbar spine, and an upturned sacrum. Hyperlordosis of the lumbar spine was also observed (Figure 2B). Electrocardiographic analysis revealed sinus bradycardia. Cardiac color Doppler ultrasonography showed no abnormalities (in 2022). The MLPA results (in 2015) also pointed to no deletions or duplications in the *DMD* gene. WES (in 2018) performed at another hospital showed negative results.

### OGM identified D4Z4 repeats and the 4qA haplotype in the family

**Figure 3:**
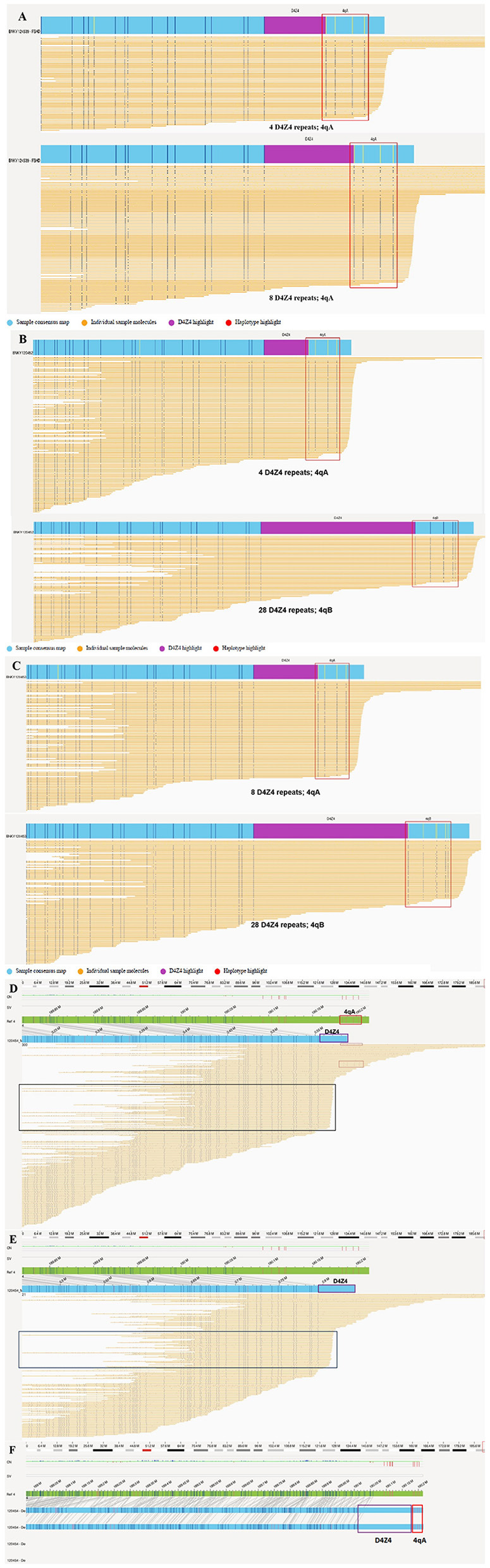
OGM results for this family. **(A)** The proband (III-9) was identified as a compound heterozygote for the 4qA allele with four and eight D4Z4 repeat units. **(B)** The younger brother (III-10) was found to only have one 4qA allele with four D4Z4 repeats and a 4qB allele with 28 D4Z4 repeat units. **(C)** The proband's father (II-7) carried the 4qA allele with eight D4Z4 repeats and a 4qB allele with 28 D4Z4 repeat units. **(D–F)** The OGM results of the proband's mother (II-8). The green bar is the reference map of chromosome 4, and the blue bar is the assembled map of chromosome 4. **(D)** Chromosome 4 of subject II-8 was found to have a calculated repeat count of ≥5 with an unknown haplotype via FSHD pipeline analysis. A high proportion of consistently truncated sequences (black boxes) might reflect terminal DNA deletions or insufficiently long DNA strands. Several sequences are followed by the 4qA haplotype (red box) with a D4Z4 array length (approximately four repeat units) shorter than that in each map containing five units (purple box). **(E)** Chromosome 4 of a subject whose haplotype was unknown had a calculated repeat count of ≥8, as determined by FSHD pipeline analysis. **(F)** A 4qA allele and 49 D4Z4 repeat units were manually determined based on the consensus maps of the de novo pipeline analysis.

WES of genomic DNA samples from all four family members did not reveal any pathogenic or likely pathogenic variations associated with the disease phenotype. But OGM analysis of all four individuals showed a repetition of D4Z4 units for both the 4qA and 4qB alleles, enabling precise determination of their genotypes (Figure 3). The proband (III-9) was a compound heterozygote for the 4qA allele with four and eight separate D4Z4 repeat units (Figure 3A), as confirmed through haplotype analysis, whereas her affected younger brother (III-10) was a heterozygote who inherited only one 4qA allele with four D4Z4 repeat units (Figure 3B). The clinical symptoms in the younger brother were more severe than those of the proband. The proband's father (II-7, aged 50 years), who was found to have a normal phenotype upon neurological examination, also carried the 4qA allele with eight D4Z4 repeat units (Figure 3C). It should be emphasized that the unaffected mother (II-8) had a 4qA allele with 49 D4Z4 repeat units and low-level mosaicism with four D4Z4 repeat units (Figures 3D, F), but the proportion of low-level mosaicism could not be confirmed. The FSHD pipeline was used for an alignment analysis to assess the support for a given map. In the chromosome 4 D4Z4 regions, the map was truncated before the ends of the repeat units, and the haplotype was considered unknown because the sequences did not fully span the repeat units and haplotype-specific labels; thus, the pipeline generated lower-bound estimates of the repeat counts of ≥5 and ≥8 (Figures 3D, E). To determine the exact number of repeats, we prepared a standard *de novo* whole-genome assembly and obtained a fully assembled map of the chromosome 4 D4Z4 region. The similarity between the intervals of the reference haplotype-specific and assembled maps was checked manually, and the homozygous haplotype 4qA was found in the unaffected mother. Repeat unit lengths were estimated based on the interval between the labels flanking the D4Z4 arrays, and the number of D4Z4 repeat units was manually calculated to be 49 for the mother (Figure 3F). Furthermore, when we manually checked the sequences supporting a given map, we found several sequences followed by the 4qA haplotype (Figure 3D [red box]) with a D4Z4 array length to be shorter than that of the given map (Figure 3D [purple box]). In addition, a large fraction of sequences was consistently truncated (Figure 3D [black boxes]), which could have been due to terminal deletion or an insufficient DNA sequence length. In addition, OGM accurately identified the D4Z4 repeat units in both the 10qA and 10qB alleles in all family members, as shown in Supplementary Figure 2.

### CpG methylation analysis

We analyzed 10 CpG sequences and used CpG6 methylation values < 73% as the threshold for FSHD according to previous literature (17). Methylation analysis showed that CpG6 methylation levels in four subjects (III-9, III-10, II-7 and II-8) were 60%, 20%, 90%, and 90%, respectively (Supplementary Table 1, Supplementary Figure 3).

Methylation levels of all the 10 CpG sequences were lower in symptomatic patients (III-9 and III-10) compared to those in asymptomatic parents (II-7 and II-8). The CpG6 methylation levels in both father (asymptomatic carrier) and mother (mosaic carrier) was greater than 73%. However, the younger brother who carried only one D4Z4 reduced allele had lower methylation levels of CpG6 (20% vs. 60%), higher CS (9 vs. 8), and higher ACCS scores (533 vs. 353) compared to the older sister who carried two D4Z4 reduced alleles (Supplementary Table 1).

## Discussion

The molecular diagnosis of FSHD is difficult because of the relatively large size of the repeat units (3.3 kb), the variable number of repeat units, the existence of homologous polymorphic repeat arrays on chromosomes 4 and 10, and potential exchanges between these chromosomal regions (18). FSHD is traditionally diagnosed by Southern blotting (8), molecular combing (19, 20), Pacific Biosciences (PacBio) sequencing (21), and Nanopore sequencing (22). The most commonly used technique for diagnosing FSHD1 is Southern blotting, which is time-consuming (because it involves multiple enzymatic reactions) and only results in an estimate of the number of D4Z4 repeat units based on the band size. In recent years, OGM has emerged as an effective method for a precise determination of the number of repeat units and differentiating DNA fragments from 4q35 and 10q26 (11, 18).

In this study, we applied OGM for the genetic diagnosis of FSHD1 in a family with two siblings who presented the classical FSHD phenotype. We found that suspected mosaicism in maternal germ cells and phenotype heterogeneity were prominent features in the family. The proband was a compound heterozygote carrying two D4Z4 alleles with four and eight 4qA-D4Z4 repeat units, and the younger brother carried a single shortened D4Z4 allele with four 4qA repeat units. The younger brother had an earlier onset of the disease at the age of 13 and higher ACCS scores than the older sister, although the proband had two D4Z4 alleles with four and eight 4qA repeats. Thus, a further methylation analysis is required to explain clinical heterogeneity in this family.

The existence of an inverse relationship between the number of D4Z4 repeats and disease severity has been confirmed in several FSHD genotype–phenotype correlation studies. In general, shortened D4Z4 alleles with 1–3 units cause more severe disease symptoms, whereas D4Z4 alleles with 4–8 units point to the classic form of FSHD (23, 24). The phenotypic expression of individuals who carried one reduced D4Z4 allele with 7–8 units was quite similar to those with 9–10 units (25). Even within families, individuals carrying the same fragment size of the repeat units have presented significant clinical variations, ranging from seriously impacted to asymptomatic individuals. Comprehending the clinical diversity of FSHD continues to be challenging. In this study, both siblings carried fragments of the same size, that is, four D4Z4 units; however, the clinical phenotype of the younger brother was more severe than that of the proband.

Wohlgemuth et al. (26) proposed that the dosage effect caused by contraction of the D4Z4 repeat array on both 4q35 chromosomes results in the compound heterozygote phenotype in probands and that disease penetrance appears to be associated with the residual repeat size. However, we observed that the clinical severity of the compound heterozygote proband carrying a paternally inherited D4Z4 allele (eight repeat units) and a maternally inherited D4Z4 allele (four repeat units) was milder than that of her younger brother, who carried a single D4Z4 allele with four repeat units from his mother. Methylation analysis showed that the compound heterozygous sister had a higher methylation level of CpG6 than the heterozygous brother, which is consistent with the conclusion of previous studies that, besides the size of the D4Z4 repeats, the level of CpG6 methylation can be used as an indicator of the severity of the disease (27).

Recent genetic data indicate that the disease penetrance remains incomplete even in older individuals, with non-penetrance rates ranging from 32% to 53%. Both the number of repeats and the extent of kinship contribute to this variation in disease penetrance (28–31). For carriers possessing eight repeat units, there exists a 20% likelihood of experiencing symptoms by the age of 70, with only a 24% probability of the disease onset later being identified through clinical examination at 30 years of age (30). However, Ruggiero et al. reported that most individuals (52.8%) carrying D4Z4 with seven to eight repeat units presented with no muscle weakness (25). The clinical phenotypic variation in FSHD is extensive, including incomplete penetrance and significant heterogeneity between individuals and within families. Interestingly, the father, who carried a D4Z4 allele with eight 4qA repeat units, did not show FSHD symptoms at 50 years (when the disease usually manifests). These conditions differ from the typical clinical manifestations of FSHD. Other studies have previously proposed that the degree of hypomethylation at distal D4Z4 arrays determines disease penetrance, and our data show the father, despite possessing hypermethylated state, with the D4Z4 methylation levels at 90%, there was no disease penetrance. Therefore, we consider the D4Z4 allele of the father as a non-penetrant allele. This not only explains why the father carried the contracted D4Z4 allele without phenotype but also why the two contracted D4Z4 repeats did not result in a more severe phenotype in our proband, which could be because the dosage effect of a “non-penetrant” 4qA allele and a “normal penetrant” 4qA allele was not increased.

Women commonly experience a later FSHD onset and exhibit atypical or milder phenotypes, the reasons for which are currently unclear (32). However, male-specific hormones such as testosterone (a powerful anabolic agent that promotes muscle protein synthesis) and muscle regeneration likely render males significantly more sensitive to the pathogenic mechanisms of FSHD (33). Additionally, because of differences in hormonal profiles, men and women may react to catabolic conditions in various ways (34, 35). Other studies showed that the different degrees of methylation levels between asymptomatic carriers and patients with FSHD1 might be caused by sex of the individuals (36). However, these hypotheses need to be confirmed in future studies.

Interestingly, among 107 individuals evaluated by Padberg (37), the proportion of asymptomatic females (21/48, 44%) was twice as high as that of males (13/59, 22%). Ricci et al. (14) noted that six of seven non-penetrant carriers in their series were female. In addition, Van der Maarel et al. (38) noted a sex difference in the presence of mosaicism and found in a review of 35 de novo FSHD families that somatic mosaicism was present in 40% of the cases, either in the patient or the asymptomatic parent. Interestingly, while mosaic males often had symptoms, mosaic females did not. According to Goto et al., the clinical symptoms of parents with mosaicism were not significantly different between the males and females despite the fact that asymptomatic female carriers with mosaicism have been reported to be more common (28, 39, 40).

This case study presents an intriguing example where post-zygotic mosaicism was inferred in the proband's mother, who was suspected of having germline mosaicism. Of all reported sporadic cases, 19% having a mosaic germline are a phenotypically normal parent, and mothers of sporadic cases may be at a higher risk of being mosaicism carriers with a higher recurrence risk for further affected children (28). In the family in our case study, the mother carried a 4qA-D4Z4 allele of normal size (49 D4Z4 units) and a low-level mosaic (in the peripheral blood) with a 4qA-D4Z4 allele (four D4Z4 units). The two siblings each carried a 4qA-D4Z4 allele of four units as well as a paternal 4qA-D4Z4 allele with eight and 28 repeat units, respectively. Given the fact that both siblings carry the 4qA allele with four D4Z4 repeat units, we speculate that the mother has a mosaic germline and that a low proportion of mosaic variants with four D4Z4 repeats and the 4qA haplotype are present in her peripheral blood cells. Therefore, we infer the absence of symptoms in the mother to be associated with low-level mosaicism and D4Z4 hypermethylation.

These findings support a previously reported observation that many parents with germline mosaicism during oogenesis may have been overlooked (40). Additionally, 15–20% of healthy parents of patients with FSHD had somatic mosaicism in the 4q35 region (38, 40–42). Lemmers et al. (43) demonstrated that the standard diagnostic techniques failed to detect somatic mosaicism in patients with FSHD. A thorough investigation of somatic and germline mosaicism in families with de novo FSHD needs to be undertaken to generate more accurate data so as to give genetic counseling to them. In this study, we manually checked the sequences that supported the OGM map.

We extended our surveys to the grandparents, the siblings of the parents, as well as their sons and daughters. None of these individuals we studied displayed any clinical phenotypes, despite many being older than the typical age of onset (over forty years old) (Figure 1A). Unfortunately, we did not collect samples from them and therefore are unable to determine whether they carry FSHD-sized alleles and possess specific methylation levels.

However, we acknowledge some limitations in our approach. The mother's 4qA-D4Z4 allele with 49 repeat units was not conducive to accurate automated analysis, and manual analysis was necessary in this case. We could not accurately determine the proportion of low-level mosaic variants with four D4Z4 repeats with the 4qA haplotype. Thus, establishing the diagnosis necessitates close exchange between clinicians and molecular geneticists regarding the genotypes and phenotypes of the subjects, as well as the exploitation of all OGM data and interpretation strategies to avoid missed diagnoses. Although the current price of OGM is relatively high, it is expected to gradually become affordable, given the ongoing commercial development and promotion of OGM technology. Despite its high cost, we believe that OGM presents a viable approach for the fast and accurate analysis of FSHD1 and also for the detection of somatic mosaicism.

## Conclusion

In conclusion, we employed the OGM approach to accurately diagnose FSHD1 in two siblings in a family, thus pointing the usefulness of OGM in the accurate genetic diagnosis of FSHD1 and to effectively exclude interference by 10q26 repeat elements. OGM also revealed low-level mosaicism in the mother's peripheral blood sample; thus, we highly suspected maternal germline mosaicism in this family. The results of this study emphasize the complexity of the genetics underlying FSHD1; high clinical variability was observed among patients carrying the same alleles, even within the same family, which could be attributed to the CpG6 methylation levels. An accurate diagnosis can aid in the genetic counseling for cases falling within the borderline range of 8–10 D4Z4 repeat units. Clinicians must also acknowledge the limitations associated with genetic testing within this borderline range. Solely relying on the number of D4Z4 repeat units is insufficient to ascertain the severity of FSHD or to determine whether these repeat units are pathogenic. Additional markers are necessary to confirm the diagnosis of FSHD.

## Data availability statement

The datasets presented in this article are not readily available because of ethical and privacy restrictions. Requests to access the datasets should be directed to the corresponding author.

## Ethics statement

The studies involving human participants were reviewed and approved by the Medical Ethics Committee of West China Second University Hospital, Sichuan University. The patients/participants provided their written informed consent to participate in this study. Written informed consent was obtained from the individual(s) for the publication of any potentially identifiable images or data included in this article.

## Author contributions

JJ: Data curation, Formal analysis, Investigation, Methodology, Writing – original draft. XC: Data curation, Formal analysis, Investigation, Writing – review & editing. HQ: Formal analysis, Investigation, Writing – review & editing. QY: Formal analysis, Investigation, Writing – review & editing. TH: Methodology, Resources, Writing – review & editing. MY: Formal analysis, Writing – review & editing. HZ: Formal analysis, Writing – review & editing. XZ: Conceptualization, Funding acquisition, Investigation, Supervision, Writing – review & editing, Writing – original draft.

## References

[1] Deenen JC Arnts H van der Maarel SM Pedberg GW Verschuuren J Bakker E. Population-based incidence and prevalence of facioscapulohumeral dystrophy. Neurology. (2014). 83:1056-9. 10.1212/WNL.0000000000000797 25122204 PMC4166358

[2] DeSimone AM Pakula A Lek A Emerson CP. Facioscapulohumeral muscular dystrophy. Compr Physiol. (2017) 7:1229–79. 10.1002/cphy.c160039 28915324

[3] Wijmenga C Hewitt JE Sandkuijl LA Clark LN Wright J Dauwerse HG. Chromosome 4q DNA rearrangements associated with facioscapulohumeral muscular dystrophy. Nat Genet. (1992) 2:26–30. 10.1038/ng0992-26 1363881

[4] Lemmers RJ van der Vliet PJ Klooster R Sacconi S Camani P Dauwerse JG. A unifying genetic model for facioscapulohumeral muscular dystrophy. Science. (2010) 329:1650–3. 10.1126/science.1189044 20724583 PMC4677822

[5] Wijmenga C Frants RR Hewitt JE van Deutekom JC van Geel M Wright TJ. Molecular genetics of facioscapulohumeral muscular dystrophy. Neuromuscul Disord. (1993) 3:487–91. 10.1016/0960-8966(93)90102-P 8186699

[6] Lemmers RJLF Tawil R Petek LM Balog J Block GJ Santen GWE. Digenic inheritance of an SMCHD1 mutation and an FSHD-permissive D4Z4 allele causes facioscapulohumeral muscular dystrophy type 2. Nat Genet. (2012) 44:1370–4. 10.1038/ng.2454 23143600 PMC3671095

[7] van den Boogaard ML Lemmers RJLF Balog J Wohlgemuth M Auranen M Mitsuhashi S. Mutations in DNMT3B modify epigenetic repression of the d4z4 repeat and the penetrance of facioscapulohumeral dystrophy. Am J Hum Genet. (2016) 98:1020–9. 10.1016/j.ajhg.2016.03.013 27153398 PMC4863565

[8] L RJL de Kievit P van Geel M van der Wielen MJ Bakker E Padberg GW. Complete allele information in the diagnosis of facioscapulohumeral muscular dystrophy by triple DNA analysis. Ann Neurol. (2001) 50:816–9. 10.1002/ana.10057 11761483

[9] Zampatti S Colantoni L Strafella C Galota RM Caputo V Campoli G. Facioscapulohumeral muscular dystrophy (FSHD) molecular diagnosis: from traditional technology to the NGS era. Neurogenetics. (2019) 20:57–64. 10.1007/s10048-019-00575-4 30911870

[10] Montagnese F de Valle K Lemmers RJLF. 268th ENMC workshop participants. 268th ENMC workshop - Genetic diagnosis, clinical classification, outcome measures, and biomarkers in Facioscapulohumeral Muscular Dystrophy (FSHD): Relevance for clinical trials. Neuromuscul Disord. (2023) 33:447–62. 10.1016/j.nmd.2023.04.005 37099914

[11] Koppikar P Shenoy S Guruju N Hegde M. Testing for facioscapulohumeral muscular dystrophy with optical genome mapping. Curr Protoc. (2023) 3:e629. 10.1002/cpz1.629 36648278

[12] Stence AA Thomason JG Pruessner JA Sompallae RR Snow A Ma D. Validation of optical genome mapping for the molecular diagnosis of facioscapulohumeral muscular dystrophy. J Mol Diagn. (2021) 23:1506–14. 10.1016/j.jmoldx.2021.07.021 34384893 PMC8647435

[13] de Greef JC Wohlgemuth M Chan OA Hansson KB Frants S Weemaes CM. Hypomethylation is restricted to theD4Z4 repeat array in phenotypic FSHD. Neurology. (2007) 69:1018–26. 10.1212/01.wnl.0000271391.44352.fe 17785671

[14] Ricci E Galluzzi G Deidda G Cacurri S Colantoni L Merico B. Progress in the molecular diagnosis of facioscapulohumeral muscular dystrophy and correlation between the number of KpnI repeats at the 4q35 locus and clinical phenotype. Ann Neurol. (1999) 45:751–7. 10360767 10.1002/1531-8249(199906)45:6<751::aid-ana9>3.0.co;2-m

[15] van Overveld PG Enthoven L Ricci E Rossi M Felicetti L Jeanpierre M. Variable hypomethylation of D4Z4 in facioscapulohumeral muscular dystrophy. Ann Neurol. (2005) 58:569–76. 10.1002/ana.20625 16178028

[16] Bionano Genomics. Bionano Solve Theory of Operation: Bionano EnFocusTM FSHD Analysis. San Diego, CA: Bionano Genomics. (2021).

[17] Calandra P Cascino I Lemmers RJ Galluzzi G Teveroni E Monoforte M. Allele-specific DNA hypomethylation characterises FSHD1 and FSHD2. J Med Genet. (2016) 53:348–55. 10.1136/jmedgenet-2015-103436 26831754

[18] Dai Y Li P Wang Z Liang F Yang F Fang L. Single-molecule optical mapping enables quantitative measurement of D4Z4 repeats in facioscapulohumeral muscular dystrophy (FSHD). J Med Genet. (2020) 57:109–20. 10.1136/jmedgenet-2019-106078 31506324 PMC7029236

[19] Nguyen K Puppo F Roche S Gaillard M Chaix C Lagarde A. Molecular combing reveals complex 4q35 rearrangements in Facioscapulohumeral dystrophy. Hum Mutat. (2017) 38:1432–41. 10.1002/humu.23304 28744936

[20] Vasale J Boyar F Jocson M Sulcova V Chan P Liaquat K. Molecular combing compared to Southern blot for measuring D4Z4 contractions in FSHD. Neuromuscul Disord. (2015) 25:945–51. 10.1016/j.nmd.2015.08.008 26420234

[21] Morioka MS Kitazume M Osaki K Wood J Tanaka Y. Filling in the gap of human chromosome 4: single molecule real time sequencing of macrosatellite repeats in the facioscapulohumeral muscular dystrophy locus. PLoS ONE. (2016) 11:e0151963. 10.1371/journal.pone.0151963 27002334 PMC4803325

[22] Mitsuhashi S Nakagawa S Takahashi Ueda M Imanishe T Frith M Mitsuhashi H. Nanopore-based single molecule sequencing of the D4Z4 array responsible for facioscapulohumeral muscular dystrophy. Sci Rep. (2017) 7:14789. 10.1038/s41598-017-13712-6 29093467 PMC5665936

[23] Nikolic A Ricci G Sera F Bucci E Govi M Mele F. Clinical expression of facioscapulohumeral muscular dystrophy in carriers of 1-3 D4Z4 reduced alleles: experience of the FSHD Italian National Registry. BMJ Open. (2016) 6:e007798. 10.1136/bmjopen-2015-007798 26733561 PMC4716236

[24] Statland JM Donlin-Smith CM Tapscott SJ Lemmers RJLF van der Maarel SM Tawil R. Milder phenotype in facioscapulohumeral dystrophy with 7-10 residual D4Z4 repeats. Neurology. (2015) 85:2147–50. 10.1212/WNL.0000000000002217 26561289 PMC4691686

[25] Ruggiero L Mele F Manganelli F Bruzzese D Ricci G Vercelli L. Phenotypic variability among patients with d4z4 reduced allele facioscapulohumeral muscular dystrophy. JAMA Netw Open. (2020) 3:e204040. 10.1001/jamanetworkopen.2020.4040 32356886 PMC7195625

[26] Wohlgemuth M Lemmers RJ van der Kooi EL Wielen MJv van Overveld PG Dauwerse H. Possible phenotypic dosage effect in patients compound heterozygous for FSHD-sized 4q35 alleles. Neurology. (2003) 61:909–13. 10.1212/WNL.61.7.909 14557558

[27] Zheng F Qiu L Chen L Zheng Y Lin X He J. Association of 4qA-specific distal D4Z4 hypomethylation with disease severity and progression in facioscapulohumeral muscular dystrophy. Neurology. (2023) 101:e225–37. 10.1212/WNL.0000000000207418 37225433 PMC10382269

[28] Goto K Nishino I Hayashi YK. Very low penetrance in 85 Japanese families with facioscapulohumeral muscular dystrophy 1A. J Med Genet. (2004) 41:e12. 10.1136/jmg.2003.008755 14729852 PMC1757263

[29] Tonini MMO Passos-Bueno MR Cerqueira A Matioli SR Pavanello R Zatz M. Asymptomatic carriers and gender differences in facioscapulohumeral muscular dystrophy (FSHD). Neuromuscul Disord. (2004) 14:33–8. 10.1016/j.nmd.2003.07.001 14659410

[30] Ricci G Scionti I Sera F Govi M D'Amico R Frambolli I. Large scale genotype–phenotype analyses indicate that novel prognostic tools are required for families with facioscapulohumeral muscular dystrophy. Brain. (2013) 136:3408–17. 10.1093/brain/awt226 24030947 PMC3808686

[31] Salort-Campana E Nguyen K Bernard R Jouve E Solé G Nadaj-Pakleza A. Low penetrance in facioscapulohumeral muscular dystrophy type 1 with large pathological D4Z4 alleles: a cross-sectional multicenter study. Orphanet J Rare Dis. (2015) 10:2. 10.1186/s13023-014-0218-1 25603992 PMC4320820

[32] Zatz M Marie SK Cerqueira A Vainzof M Pavanello RC Passos-Bueno MR. The facioscapulohumeral muscular dystrophy (FSHD1) gene affects males more severely and more frequently than females. Am J Med Genet. (1998) 77:155-61. 9605290

[33] Anderson LJ Liu H Garcia JM. Sex differences in muscle wasting. Adv Exp Med Biol. (2017) 1043:153–97. 10.1007/978-3-319-70178-3_9 29224095

[34] Bredella MA. Sex differences in body composition. Adv Exp Med Biol. (2017) 1043:9–27. 10.1007/978-3-319-70178-3_2 29224088

[35] Ruggiero L Manganelli F Santoro L. Muscle pain syndromes and fibromyalgia: the role of muscle biopsy. Curr Opin Support Palliat Care. (2018) 12:382–7. 10.1097/SPC.0000000000000355 29912728

[36] Gaillard M-C Roche S Dion C Tasmadjian A Bouget G Salort-Campana E. Differential DNA methylation of the D4Z4 repeat in patients with FSHD and asymptomatic carriers. Neurology. (2014) 83:733–42. 10.1212/WNL.0000000000000708 25031281

[37] Padberg. Facioscapulohumeral Disease (PhD Thesis) The Netherlands: Intercontinental Graphics, (1982) p. 243. Available online at: from https://hdl.handle.net/1887/25818

[38] van der Maarel SM Deidda G Lemmers RJ van Overveld PG van der Wielen M Hewitt JE. De novo facioscapulohumeral muscular dystrophy: frequent somatic mosaicism, sex-dependent phenotype, and the role of mitotic transchromosomal repeat interaction between chromosomes 4 and 10. Am J Hum Genet. (2000) 66:26–35. 10.1086/302730 10631134 PMC1288331

[39] Zatz M Marie SK Passos-Bueno MR Vainzof M Campiotto S Cerqueira A. High proportion of new mutations and possible anticipation in Brazilian facioscapulohumeral muscular dystrophy families. Am J Hum Genet. (1995) 56:99–105. 7825608 PMC1801310

[40] Köhler J Rupilius B Otto M Bathke K Koch MC. Germline mosaicism in 4q35 facioscapulohumeral muscular dystrophy (FSHD1A) occurring predominantly in oogenesis. Hum Genet. (1996) 98:485–90. 10.1007/s004390050244 8792827

[41] Padberg GW Frants RR Brouwer OF Wijmenga C Bakker E Sandkuiji LA. Facioscapulohumeral muscular dystrophy in the Dutch population. Muscle Nerve Suppl. (1995) 2:S81–4. 10.1002/mus.880181315 23573591

[42] Upadhyaya M Maynard J Osborn M Jardine P Harper PS Lunt P. Germinal mosaicism in facioscapulohumeral muscular dystrophy (FSHD). Muscle Nerve Suppl. (1995) 2:S45-9. 10.1002/mus.880181310 23573586

[43] Lemmers RJLF van der Wielen MJR Bakker E Padberg GW Frants RR van der Maarel SM. Somatic mosaicism in FSHD often goes undetected. Ann Neurol. (2004) 55:845–50. 10.1002/ana.20106 15174019

