# Supplementary material for: Case report: Identification of facioscapulohumeral muscular dystrophy 1 in two siblings with normal phenotypic parents using optical genome mapping
Source: Front Neurol. 2024 Feb 1;15:1258831. doi: 10.3389/fneur.2024.1258831 (PMC10867183; doi:10.3389/fneur.2024.1258831)
Supplement: Supplementary file 2 [file Presentation_1.pptx]

## Slide 1
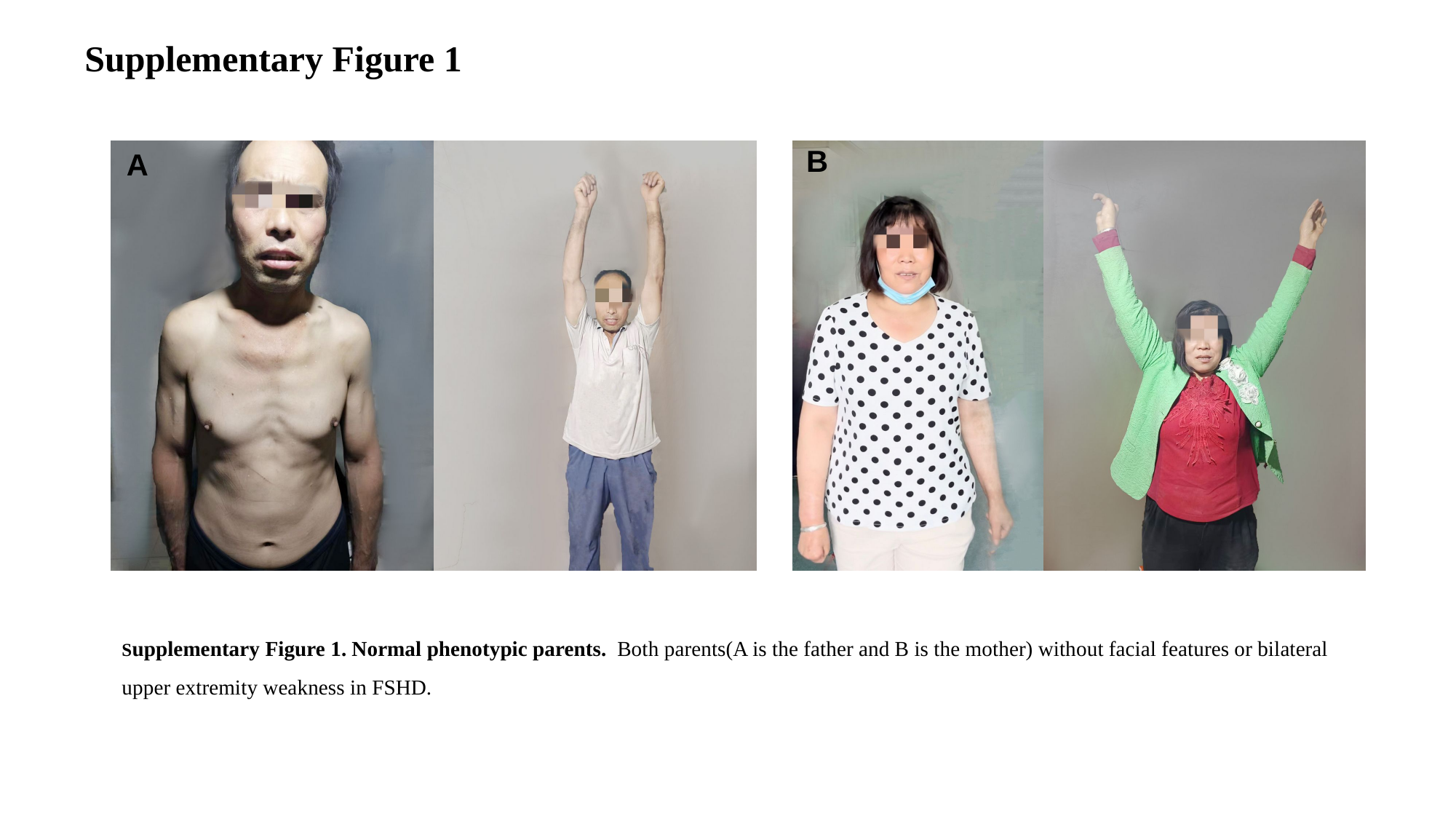

Supplementary Figure 1
B
A
Supplementary Figure 1. Normal phenotypic parents. Both parents(A is the father and B is the mother) without facial features or bilateral upper extremity weakness in FSHD.

## Slide 2
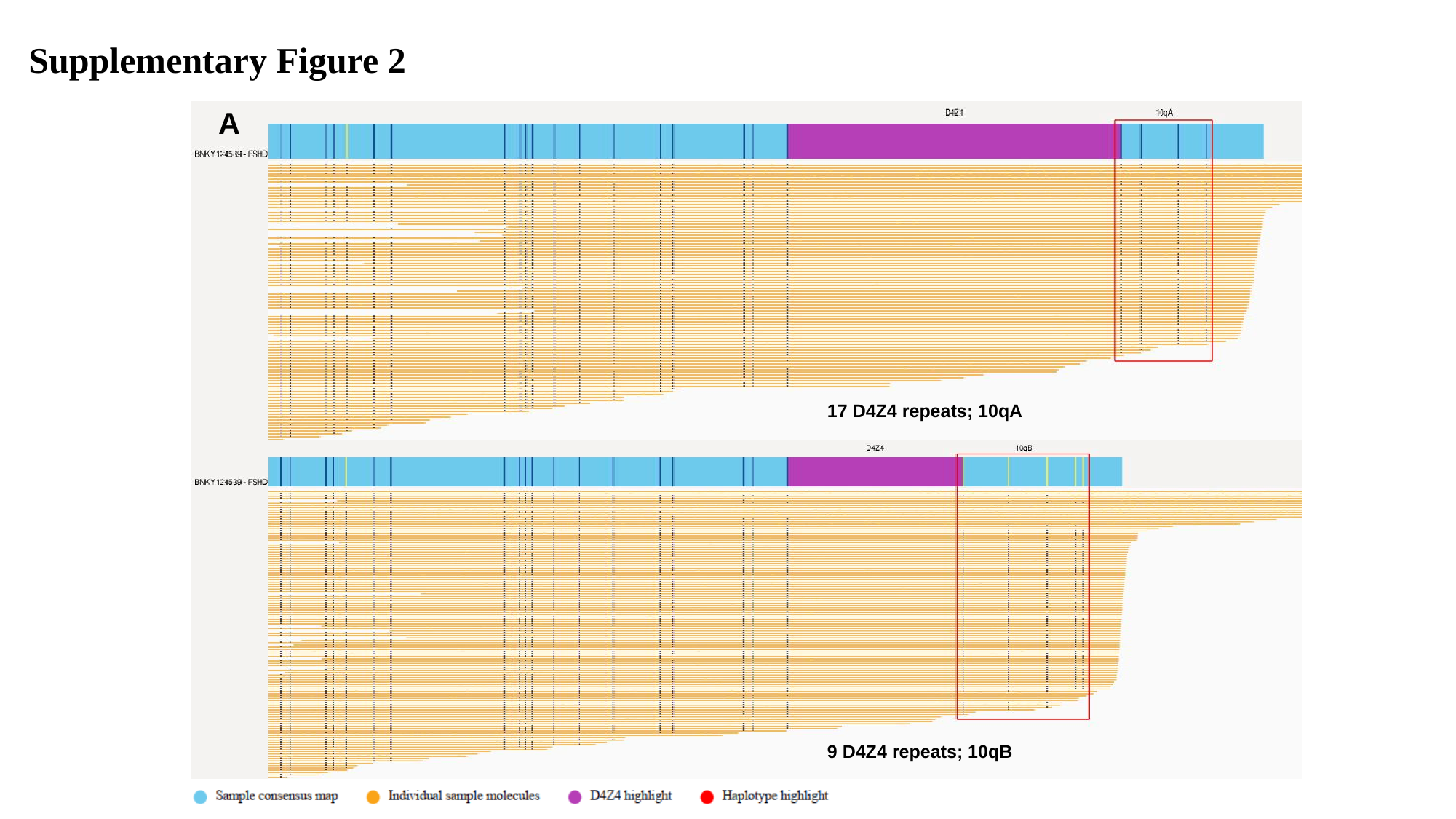

Supplementary Figure 2
A
17 D4Z4 repeats; 10qA
9 D4Z4 repeats; 10qB
17 D4Z4 repeats; 10qA
9 D4Z4 repeats; 10qB

## Slide 3
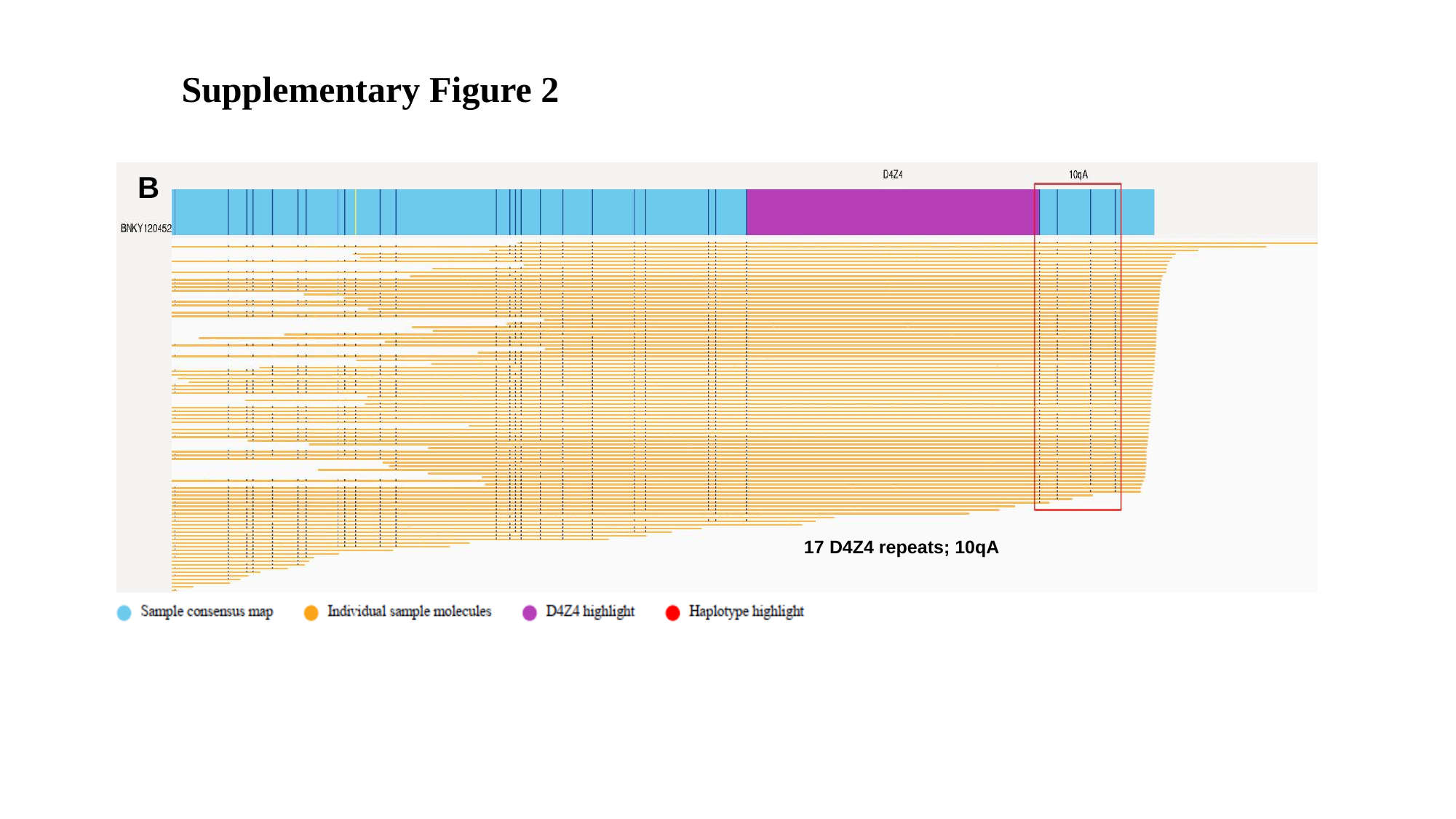

Supplementary Figure 2
B
17 D4Z4 repeats; 10qA
17 D4Z4 repeats; 10qA

## Slide 4
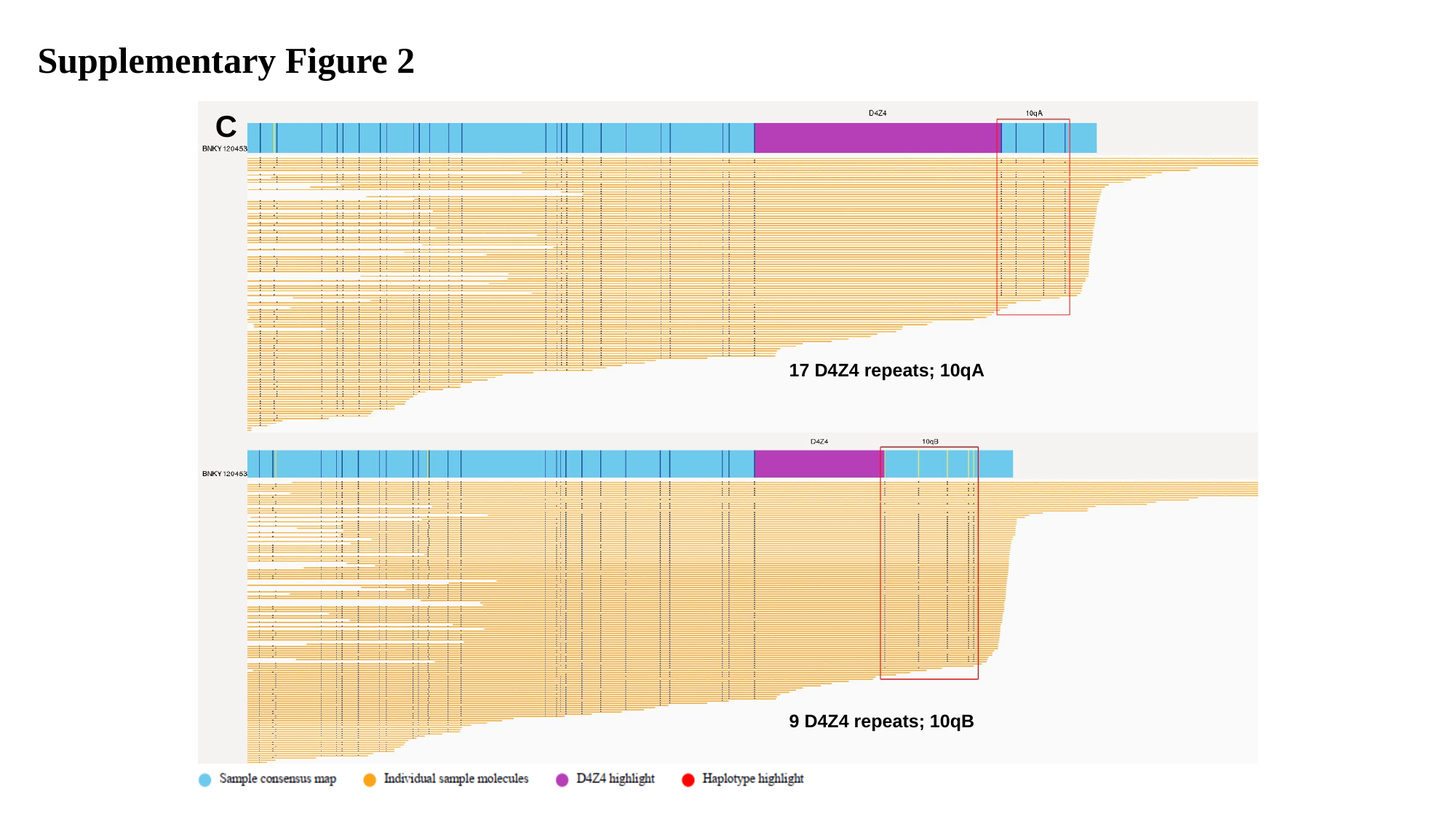

Supplementary Figure 2
17 D4Z4 repeats; 10qA
9 D4Z4 repeats; 10qB
C

## Slide 5
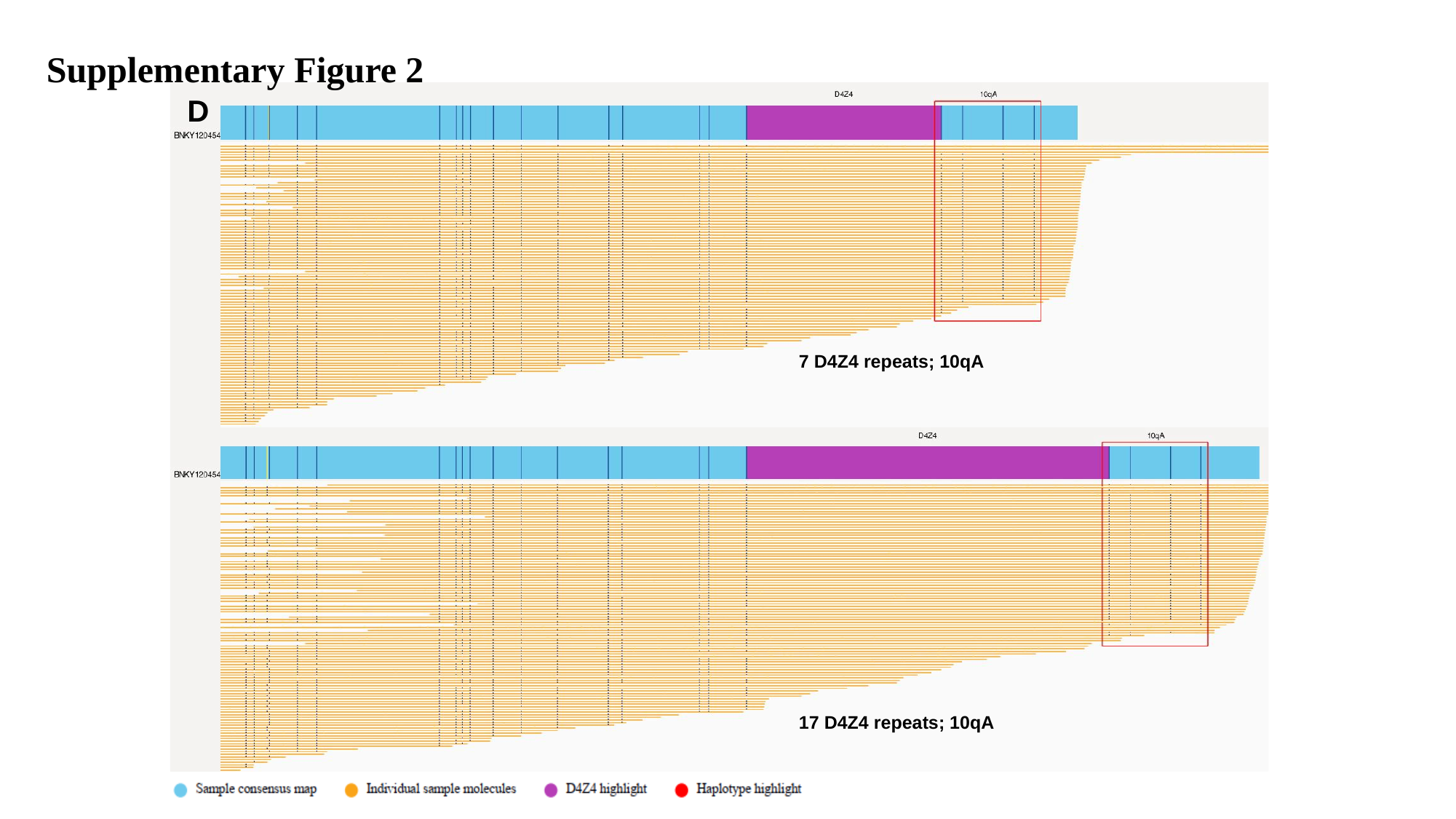

Supplementary Figure 2
D
7 D4Z4 repeats; 10qA
17 D4Z4 repeats; 10qA

## Slide 6
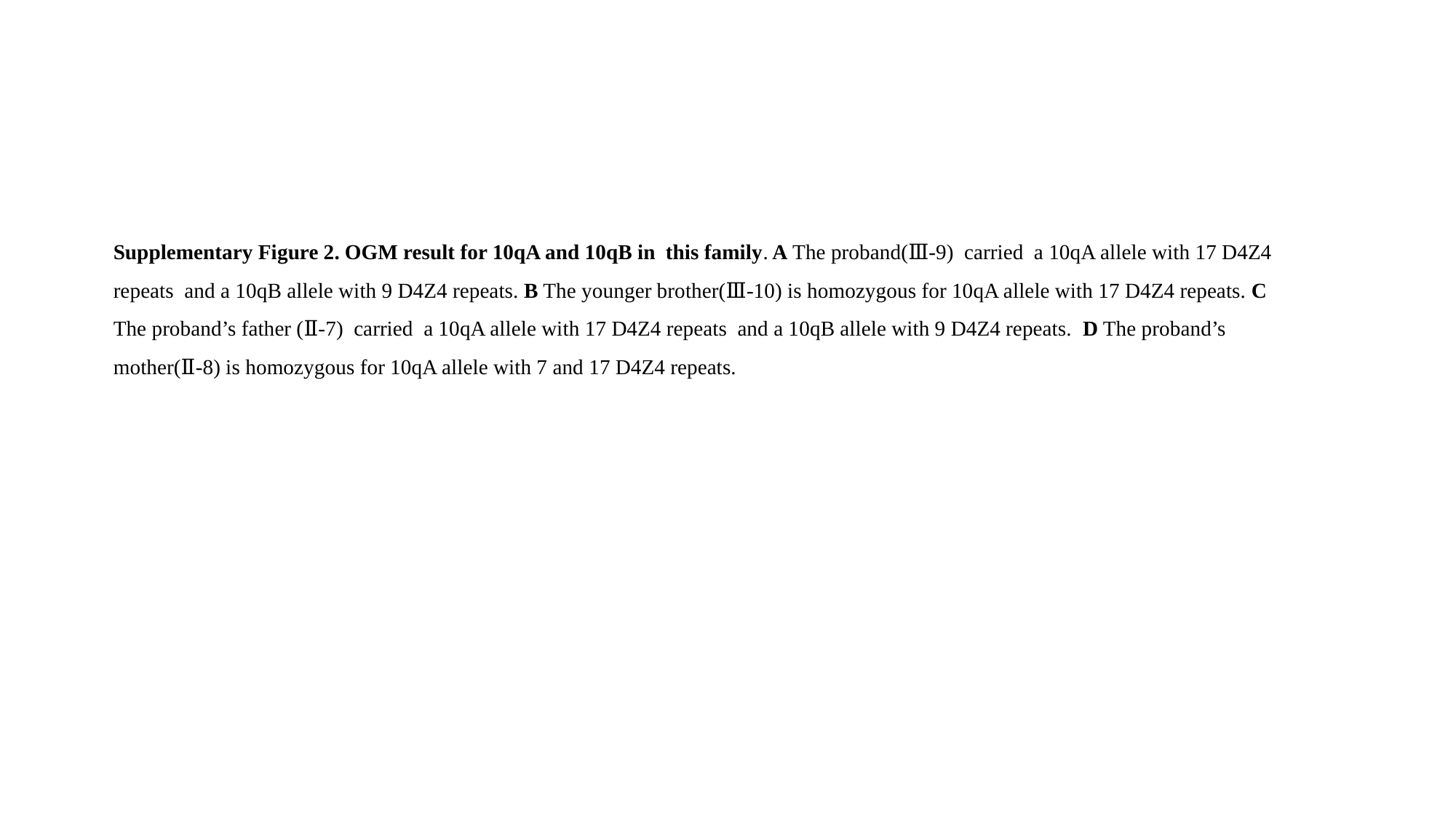

Supplementary Figure 2. OGM result for 10qA and 10qB in this family. A The proband(Ⅲ-9) carried a 10qA allele with 17 D4Z4 repeats and a 10qB allele with 9 D4Z4 repeats. B The younger brother(Ⅲ-10) is homozygous for 10qA allele with 17 D4Z4 repeats. C The proband’s father (Ⅱ-7) carried a 10qA allele with 17 D4Z4 repeats and a 10qB allele with 9 D4Z4 repeats. D The proband’s mother(Ⅱ-8) is homozygous for 10qA allele with 7 and 17 D4Z4 repeats.

## Slide 7
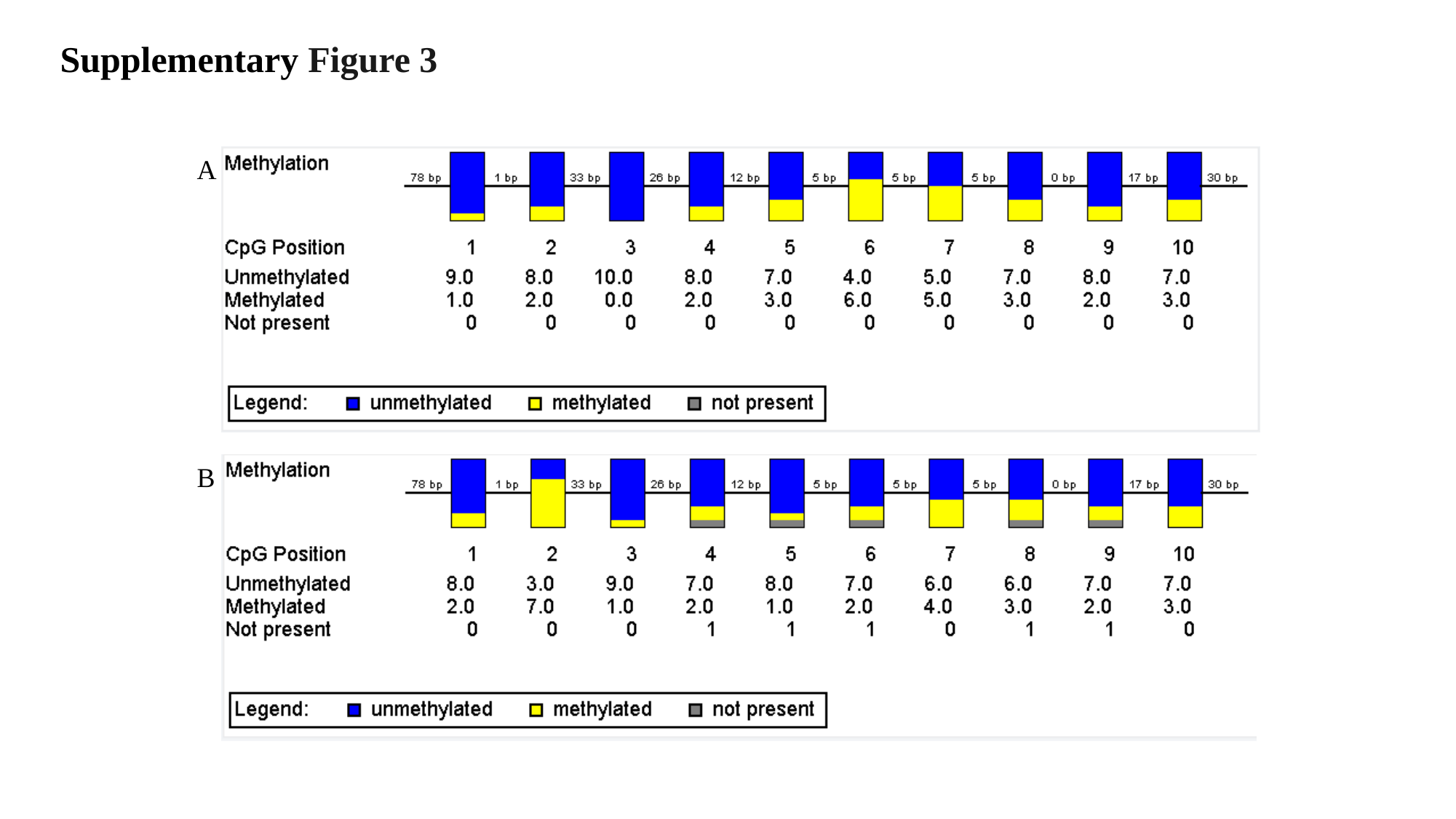

Supplementary Figure 3
A
B

## Slide 8
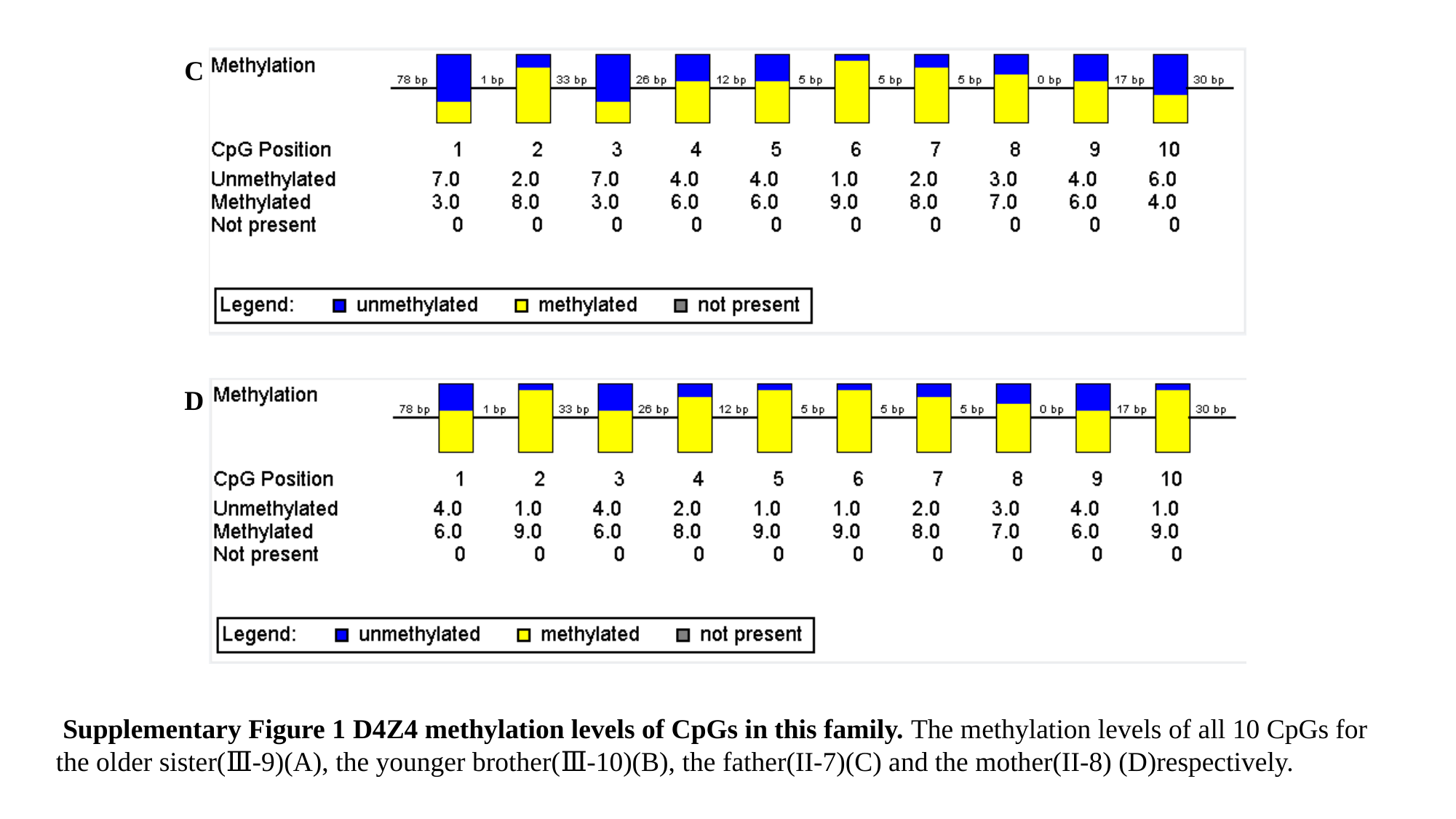

C
D
 Supplementary Figure 1 D4Z4 methylation levels of CpGs in this family. The methylation levels of all 10 CpGs for the older sister(Ⅲ-9)(A), the younger brother(Ⅲ-10)(B), the father(II-7)(C) and the mother(II-8) (D)respectively.
